# Knowledge, attitudes, and practices of Chinese operating room nurses toward isolation in oncologic surgery: a cross-sectional survey

**DOI:** 10.7717/peerj.21818

**Published:** 2026-11-09

**Authors:** Mingjiao Zhang, Xin Liao, Li Xie, Qi Yuan

**Affiliations:** 1 Key Laboratory of Birth Defects and Related Diseases of Women and Children (Sichuan University), Ministry of Education, Chengdu, Sichuan, China; 2 Department of Operating Room Nursing, West China Second University Hospital, Sichuan University, Chengdu, Sichuan Province, China

**Keywords:** Surgical isolation technique, Oncological surgery, Operating room nurses, Knowledge, attitudes, practices, Cross-sectional study

## Abstract

**Background:**

Surgical isolation techniques are a critical fundamental skill in oncological surgery, essential for preventing the dissemination and implantation of tumor cells. However, research on the knowledge, attitudes, and practices (KAP) of surgical isolation techniques among operating room nurses remains limited. This study aims to assess the KAP levels of isolation techniques among Chinese operating room nurses during oncological surgeries.

**Method:**

This single-center, cross-sectional study was conducted between October and December 2024. Operating room nurses from a tertiary hospital were recruited *via* non-probabilistic convenience sampling, yielding 121 valid responses through the Wenjuanxing online platform. A validated KAP questionnaire based on relevant guidelines was developed to assess participants’ KAP regarding surgical isolation techniques. Data were analyzed using descriptive statistics to evaluate overall KAP levels and distribution.

**Result:**

A total of 121 operating room nurses were included, comprising 18 males (14.88%) and 103 females (85.12%). Among them, 84.30% were under 40 years old, and 92.56% held a bachelor’s degree or higher. The assessment revealed that the scores for operating room nurses’ KAP (correct rate) were 69.94 ± 10.15 (69.94%), 40.95 ± 3.99, and 45.10 ± 4.46, respectively. The three lowest-scoring knowledge items were related to the principles of tumor resection, surgical incision protection, and pneumoperitoneum management during laparoscopic tumor surgery. The three lowest-scoring attitude items were: “using surgical isolation techniques to facilitate surgical progress,” “using surgical isolation techniques does not increase workload,” and “feeling satisfied with one’s own mastery of isolation techniques.” The three lowest-scoring practice items involved handling instruments that contact the tumor, handling dressings that contact the tumor, and changing gloves before closing the abdomen.

**Conclusion:**

While operating room nurses maintain positive attitudes toward isolation techniques, gaps remain in their knowledge of tumor-free manipulation, instrument handling, and zone demarcation. To standardize clinical practices during tumor surgeries, regular performance audits and targeted training programs are strongly recommended.

## Introduction

In recent years, the incidence of tumors has continued to rise (Siegel et al., 2025). Surgical resection remains the primary treatment modality for most solid tumors and aims to reduce tumor burden by removing as much tumor tissue as possible, thereby improving patient survival and cure rates (Parnham et al., 2018; Pang, Muneer & Alnajjar, 2022). During oncologic surgery, the implementation of tumor-free measures by medical staff plays a critical role in patient prognosis and tumor recurrence. Previous studies have shown that proper adherence to tumor-free techniques can significantly reduce postoperative tumor recurrence (Li Brizzi et al., 2021; Yamaguchi et al., 2020; Liu et al., 2020; Gebreslase et al., 2026). In oncological surgery, surgical isolation techniques are vital for preventing tumor dissemination (Wang, Su & Zheng, 2026; Aiolfi et al., 2021). Operating room nurses serve as the primary gatekeepers in maintaining this tumor-free barrier, playing a critical role in segregating tumor-exposed instruments and ensuring protocol compliance (Chellam Singh & Arulappan, 2023). The effective and standardized implementation of these principles depends on the specialized practices of nursing staff. As integral members of the surgical team, operating room nurses serve as the primary gatekeepers in maintaining the tumor-free barrier, play a critical role in managing tumor-exposed instruments and ensuring strict adherence to tumor isolation protocols (Møller et al., 2024), which directly influences patient outcomes and postoperative recovery (Benedetto et al., 2022). Specifically, nurses segregate instruments that have come into contact with tumor tissue, closely monitor the procedure to supply sterile replacements, and prevent contaminated instruments from being reintroduced into tumor-free zones (Von Vogelsang et al., 2020). Additionally, they ensure that tumor-exposed dressings are not reused and that separate closing instruments are prepared in advance to avoid tract implantation at the incision site (Zhu et al., 2019). Collectively, these perioperative nursing interventions are crucial in preventing mechanical tumor cell seeding and dissemination (Markström et al., 2026). Nevertheless, the application of isolation techniques often varies based on subjective interpretation, potentially increasing intraoperative tumor exposure, leading to tumor upstaging, and adversely affecting patient prognosis (Matsuda et al., 2026; Berger-Richardson et al., 2018). To address this issue, this study aims to assess the knowledge, attitudes, and practices (KAP) of operating room nurses regarding surgical isolation protocols, providing evidence to support standardized management in oncological procedures.

The 2016 *Chinese Guidelines for Operating Room Nursing Practice* first introduced the concept of surgical isolation techniques, formally incorporating “tumor-free technology” within this framework (Zhu et al., 2019). These techniques comprise a series of isolation measures rooted in aseptic principles, designed to sequester tumor cells, implanted cells, contaminated sources, and infectious agents from normal tissues to prevent shedding, implantation, and dissemination (Chen et al., 2020; Sharma et al., 2021). Consequently, strict adherence by operating room nurses is paramount to minimizing the risk of intraoperative tumor seeding and metastasis. In clinical practice, however, nurses assisting in oncologic procedures frequently rely on generalized operative principles rather than standardized, procedure-specific guidelines (Sonoiki, Young & Alexis, 2020). This implementation variability, driven by individual interpretation, potentially elevates the risk of iatrogenic tumor exposure and adversely affects long-term patient prognosis. To address these gaps, this study aims to evaluate operating room nurses’ KAP regarding isolation techniques during oncologic surgery, thereby providing an empirical foundation to support standardized protocol management.

## Materials and Methods

### Design and sample

This study was designed as a single-center cross-sectional survey conducted between October and December 2024. Operating room nurses from a tertiary hospital in Sichuan Province were recruited using a non-probabilistic convenience sampling method. The inclusion criteria were as follows: (1) currently engaged in clinical operating room nursing for at least one year; (2) possession of a valid registered nurse practice certificate issued by the National Health Commission of China; and (3) voluntary participation in this study with adequate cognitive capacity to complete the questionnaire. The exclusion criteria were: (1) external nurses participating in advanced training programs and (2) trainees undergoing standardized residency training.

### Ethics approval and informed consent

The study protocol was conducted in accordance with the Declaration of Helsinki and approved by the Medical Ethics Committee of West China Second University Hospital, Sichuan University, prior to study initiation (Approval No. 2024 (443)). Participation was voluntary and anonymous, and participants were informed that they could withdraw their consent at any time during the survey. Electronic informed consent was obtained from all participants before completion of the questionnaire. All data were collected securely stored on password-protected servers, restricted to the primary research team, and processed confidentially and anonymously.

### Questionnaire distribution and data collection

The survey was administered online *via* an electronic link using the Wen juanxing Online Platform (https://www.wjx.cn/). Eligible operating room nurses were fully informed of the study’s purpose and protocols prior to enrollment. To ensure data integrity, all questionnaire items were set as mandatory, which effectively precluded missing responses. Participants were allowed to review and modify their answers before final submission, and unique IP address tracking restricted each respondent to a single submission. Over the two-month survey period, a total of 130 operating room nurses participated. Following a quality audit, 121 questionnaires were deemed valid, yielding a high response rate of 93.07%.

### Measures

The general information section of the questionnaire collected data on participants’ age, gender, educational background, total years of work experience, years of operating room experience, and professional title.

#### Survey on isolation techniques in tumor surgery

The questionnaire was developed by the research team in accordance with the 2024 *Operating Room Nursing Practice Guidelines*. It comprised 16 items encompassing six core operational domains of surgical isolation techniques: tumor resection principles, incision protection, operating table isolation zones, instrument and dressing management, intraoperative tumor-free instrument handling, and laparoscopic pneumoperitoneum parameters (pressure and flow rates). In the knowledge dimension, each correct response scored 1 point and incorrect responses scored 0. The knowledge score rate was operationalized as the mean score divided by the maximum possible score, expressed as a percentage. The accuracy rate for individual knowledge items was calculated as the proportion of correct respondents relative to the total sample (*N* = 121).

#### Survey on attitude and practices related to surgical isolation techniques in oncology

The attitudes dimension evaluated participants’ perceptions regarding the clinical significance of isolation techniques, their awareness of proactive intraoperative execution, and the perceived value of specialized training. Responses were scored on a 5-point Likert scale, ranging from 1 (strongly disagree) to 5 (strongly agree). The practices dimension captured concrete behavioral components, including specimen collection, tumor-free management of dressings and instruments, application of irrigation solutions, and demarcation of isolation zones. These items were rated on a 5-point Likert scale from 1 (never performed) to 5 (always performed), with higher scores indicating stronger beliefs and better practices. To ensure structural and content rigor, the initial instrument was refined through two rounds of Delphi expert consultation involving 10 nursing experts. Psychometric evaluation confirmed the instrument’s robust reliability and validity, yielding an overall scale Content Validity Index (S-CVI) of 0.874 and a satisfactory Cronbach’s alpha coefficient of 0.735.

### Statistical analysis

To facilitate international comparison, participants’ professional titles were categorized into three tiers according to the Chinese nursing hierarchical system: primary titles (junior and senior professional nurses), intermediate titles (competent/charge nurses), and senior titles (associate professor of nursing and co-chief/chief superintendent nurses). Data analysis was conducted using SPSS Version 26.0 (IBM Corp., Armonk, NY, USA). Given the cross-sectional nature of this descriptive baseline survey, statistical analysis was restricted to descriptive statistics. Continuous variables (such as items and dimension scores) are presented as means ± standard deviations, whereas categorical variables (such as demographics and categorical compliance rates) are expressed as frequencies and percentages.

## Results

### KAP scores according to participant characteristics

A total of 121 operating room nurses completed the questionnaire. The mean age of the participants was 31.79 ± 7.31 years, with an age range of 22 to 54 years. Table 1 summarizes the participants’ sociodemographic characteristics and presents comparisons of KAP scores according to sample characteristics. Most participants were female (85.1%), held a bachelor’s degree (87.6%), and had junior or intermediate professional titles (95.87%). Further analysis demonstrated that attitude and practice scores differed significantly across age groups, years of work experience, and professional titles.

**Table 1:** Socio-demographic characteristics and correlations with knowledge, attitude, and practice regarding tumor surgery isolation techniques. Expressed as percentages and mean ± standard deviation.

Variables	Frequency (*n*, %)	Knowledge		Attitude		Practice	
		Mean ± SD	Test results	Mean ± SD	Test results	Mean ± SD	Test results
Gender							
Male	18 (14.88)	68.40 ± 8.43	*t* = −0.649 *p* = 0.489	40.61 ± 5.48	*t* =−0.389 *p* = 0.698	46.17 ± 3.86	*t* =1.101 *p* = 0.237
Female	103 (85.12)	70.20 ± 10.44		41.01 ± 3.71		44.91 ± 4.54	
Age (years)							
<30	54 (44.63)	68.63 ± 9.75	*F* = 1.538 *p* = 0.219	39.89 ± 4.80	*F* =3.666 *P* = 0.029	44.61 ± 4.57	*F* =0.818 *P* = 0.444
30–39	48 (39.67)	70.05 ± 9.82		41.92 ± 3.34		45.73 ± 4.12	
≥40	19 (15.70)	73.36 ± 11.76		41.53 ± 1.81		44.89 ± 5.02	
Service years							
1–9	47 (38.84)	68.88 ± 9.71	*F* = 0.819 *P* = 0.443	39.57 ± 5.14	*F* =5.317 *P* = 0.006	44.55 ± 4.65	*F* =0.577 *P* = 0.563
10–19	38 (31.40)	69.57 ± 8.98		41.42 ± 3.04		45.50 ± 4.56	
≥20	36 (29.75)	71.70 ± 11.81		42.25 ± 2.38		45.39 ± 4.16	
Professional title							
Primary	59(48.76)	68.33 ± 9.63	*F* = 2.091 *P* = 0.128	40.07 ± 4.72	*F* =3.260 *P* = 0.042	44.64 ± 4.49	*F* =5.837 *P* = 0.004
Intermediate	57(47.11)	71.05 ± 10.07		41.91 ± 3.04		46.05 ± 3.99	
Senior	5(4.13)	76.25 ± 14.92		40.40 ± 1.34		39.60 ± 5.37	
Highest education attained							
Associate degree and below	6 (4.96)	68.75 ± 7.91	*F* = 0.920 *P* = 0.401	42.50 ± 3.89	*F* =0.648 *P* = 0.525	43.83 ± 5.78	*F* =2.790 *P* = 0.066
Bachelor’s degree	106 (87.60)	69.63 ± 10.22		40.93 ± 4.13		45.43 ± 4.19	
Master’s degree and above	9 (7.43)	74.31 ± 10.57		40.11 ± 1.76		42.00 ± 5.83	

### Descriptive analysis of KAP scores

Table 2 presents the three lowest-scoring items in each dimension of the KAP Assessment Scale. After conversion to a percentage scale, the mean knowledge score was 69.94 ± 10.15 out of 100, corresponding to a correct rate of 69.94%. The lowest-scoring knowledge items were related to principles of tumor resection (0.39 ± 0.49), surgical incision protection (0.44 ± 0.50), and pneumoperitoneum management during laparoscopic tumor surgery (0.50 ± 0.50). The mean attitude score was 40.95 ± 3.99 out of a possible 50. The three lowest-scoring attitude items concerned perceptions that isolation techniques promote surgical efficiency (3.77 ± 1.42), beliefs that isolation techniques do not increase workload (3.88 ± 1.19), and self-assessed mastery of isolation techniques (4.00 ± 0.82). With respect to practices related to isolation techniques, the mean score was 45.10 ± 4.46 out of 55. The lowest-scoring practice items involved the handling of instruments that had contacted tumor tissue (1.34 ± 0.67), management of dressings contaminated by tumor contact (2.15 ± 1.20), and changing gloves prior to abdominal closure (3.37 ± 1.37).

**Table 2:** The three items with the lowest scores in each dimension of the knowledge, attitude, and practice assessment scale for operating room nurses in tumor surgery isolation techniques (*n* = 121). Expressed as mean ± standard deviation.

Dimension	Items	Scores (Mean ± SD)	Correct rate (%)
Knowledge	K2: Principles for intraoperative tumor resection	0.39 ± 0.49	54.96
	K5: Protection of surgical incisions in malignant tumor surgery	0.44 ± 0.50	57.44
	K9: Management of insufflation gas during laparoscopic surgery to reduce tumor cell seeding	0.50 ± 0.50	65.70
Attitude	A5: The application of isolation technology does not affect the surgical process.	3.77 ± 1.42	N/A
	A6: Isolating surgical instruments does not increase the workload for nurses.	3.88 ± 1.19	N/A
	A8: Satisfied with the mastery of surgical isolation techniques	4.00 ± 0.82	N/A
Practice	P5: Instruments that have come into contact with the tumor should be sterilized before reuse.	1.34 ± 0.67	1.70
	P3: After wringing out the soaked gauze pad placed inside the body cavity, it should not be reused.	2.15 ± 1.20	5.80
	P8: Before abdominal examination, remind the doctor to change gloves.	3.37 ± 1.37	28.90

## Discussion

This study assessed operating room nurses’ KAP regarding isolation techniques during oncologic surgeries. Notably, peer-reviewed empirical investigations on this specific topic remain exceptionally scarce worldwide, which constrains our ability to perform extensive horizontal comparisons. By mapping the baseline KAP of 121 nurses, this research directly fills a critical empirical gap, providing foundational data to inform targeted clinical interventions and future policy development.

### Analysis of current knowledge regarding surgical isolation techniques

The findings of this study indicate that operating room nurses achieved an overall accuracy rate of 69.94% in the knowledge section, reflecting a moderate proficiency in theoretical comprehension. In terms of demographic descriptive trends, higher knowledge scores were descriptively observed alongside longer work experience, higher professional titles, and greater educational attainment. Specifically, nurses with extended tenure demonstrated a clear tendency toward higher scores, potentially owing to cumulative clinical exposure over time (Amsalu, Messele & Adane, 2021). Similarly, individuals holding advanced professional titles exhibited relatively higher average scores, which aligns with the extensive knowledge and practical insights accumulated throughout their careers (Weng et al., 2025). Additionally, nurses possessing higher educational qualifications, such as a bachelor’s degree or above, showed a descriptive tendency toward a stronger theoretical foundation, facilitating a more comprehensive understanding of isolation protocols (Weng et al., 2025; Shu et al., 2025).

However, despite this moderate overall baseline, lower descriptive accuracy was heavily concentrated in three critical domains—tumor resection principles, surgical incision protection, and intraoperative laparoscopic pneumoperitoneum management—suggesting discrete conceptual and behavioral gaps driven by systemic and logistical factors. First, the poor mastery of tumor resection and incision protection principles may be partly attributed to the lack of globally unified operational guidelines for tumor-free techniques, a regulatory gap potentially further compounded by traditional perceptions that widely assume intraoperative tumor dissemination is predominantly influenced by the surgeon’s operative skills rather than nursing practices (Acs et al., 2022; Guerrero-Macías et al., 2026). Second, regarding the frequent reuse of instruments that have contacted tumor lesions, this sub-optimal practice is largely driven by severe clinical constraints within high-volume tertiary hospitals (Almodibeg & Smith, 2021), where heavy surgical workloads and fast-paced workflows are often bottlenecked by a limited stock of precision instruments, making it logistically and financially impractical to equip two distinct sets for every oncologic operation (Eussen et al., 2025; Nast & Swords, 2019). Third, the deficit in laparoscopic pneumoperitoneum parameter management reflects a critical training gap, wherein nurses lack sufficient understanding regarding the underlying biophysical mechanisms of gas-driven aerosolization and subsequent tumor seeding (Benabou et al., 2021; Wu, Li & Zheng, 2023).

These multidimensional vulnerabilities underscore that hospital administrators must move beyond generic lectures and implement multi-faceted, targeted interventions that directly address these protocol, resource, and pedagogical gaps. Locally, to counteract the lack of standardized guidance and the traditional misconception of nursing irrelevance, hospital leadership should spearhead the formulation of procedurally explicit operating specifications that clearly define the collaborative role of nursing staff in preventing oncology seeding (Richardson et al., 2026). Furthermore, to resolve the logistical bottlenecks of heavy workloads and restricted instrument inventory, nursing managers must optimize operational scheduling and advocate for systemic logistical support, such as streamlining rapid-turnover sterilization pathways and internal resource allocation (Boamah et al., 2026; Kiwanuka et al., 2026).

Finally, to bridge the interdisciplinary knowledge deficit in pneumoperitoneum dynamics, implementing diverse, interactive training programs—particularly perioperative *in-situ* simulation and multidisciplinary team (MDT) scenario-based training—represents an effective strategy for enhancing theoretical retention and clinical compliance (Altinbas et al., 2025; Blaak et al., 2025). For instance, Guerrero et al. (2024) demonstrated that high-fidelity and virtual simulation training significantly bolster professional competence, yielding superior knowledge and technical skill acquisition. Consequently, tailoring these integrated management and training pathways to align with nurses’ diverse professional profiles, while routinely incorporating guideline dissemination and joint clinical rounds, is strongly warranted to effectively standardize oncologic isolation protocols and safeguard patient prognosis.

### Analysis of current attitude toward surgical isolation techniques

Operating room nurses generally demonstrated a positive attitude toward surgical isolation techniques, reflecting a robust recognition of their clinical significance in oncological procedures. However, an analysis of the three lowest-scoring items within this dimension revealed that nurses frequently perceive the execution of these protocols as an impediment that delays surgical pacing and inflates workloads, while concurrently expressing dissatisfaction with their own technical mastery. These unfavorable perceptions could be influenced by several clinical realities. During complex oncology cases, scrub nurses bear the dual burden of monitoring intraoperative progression and rigorously segregating (Aholaakko, 2011), tracking, and processing tumor-free *versus* tumor-affected instruments and dressings (Tjugum et al., 2026). In fast-paced, high-volume surgical environments, these overlapping tasks can easily overwhelm nursing staff, compounding physical fatigue and work-related stress (Zhai et al., 2025; Wei et al., 2023). Furthermore, insufficient protocol mastery may erode procedural confidence; when working under perceived time pressure from the surgical team, nurses might inadvertently deprioritize isolation steps, thereby compromising execution fidelity (Rahmani et al., 2025).

To mitigate these workflow and psychological bottlenecks, hospital administration should implement reality-tested operational protocols and establish structured perioperative briefings alongside interdisciplinary communication mechanisms to alleviate perceived interpersonal pressure between nurses and the surgical team (Sarwani, Sulaiman & O’Keeffe, 2025; Mann et al., 2026). Concurrently, institutional logistics should be optimized to expand the inventory of sterile surgical kits specifically designated for tumor-positive and tumor-negative fields, directly alleviating the intraoperative processing burden on scrub nurses. Ultimately, standardizing these clinical workflows within a supportive organizational climate not only optimizes practical execution but also sustainably enhances nurses’ long-term knowledge, attitudes, and compliance (Zare et al., 2025).

### Analysis of current practices regarding surgical isolation techniques

Operating room nurses demonstrated moderate-to-high scores in surgical isolation behaviors, indicating acceptable routine execution across general perioperative environments. However, relatively lower compliance was observed in three critical practice domains: the standardized management of instruments and dressings contaminated by tumor tissue, the establishment of surgical incision protection barriers, and the mandatory changing of gloves prior to abdominal closure. According to the KAP theoretical framework, comprehensive theoretical comprehension serves as the requisite cognitive foundation for sustained behavioral modification (Lu et al., 2024; Li, Wang & Jian, 2025); hence, these practical deficiencies might be closely linked to the conceptual blind spots identified in the knowledge dimension. To effectively rectify these behavioral gaps, hospital administrators should design highly contextualized training curricula tailored to distinct surgical sub-specialties and operative approaches. For instance, the operational requirements for maintaining a tumor-free field vary drastically between open and minimally invasive surgeries. Prior evidence indicates that laparoscopic tumor procedures, by virtue of their specific gas-driven environment, are uniquely susceptible to port-site metastasis or puncture-hole seeding compared to conventional open approaches (Gao, Guo & Wang, 2020). Consequently, targeted operational interventions must explicitly address advanced intraoperative variables—such as the meticulous regulation of pneumoperitoneal pressure, gas flow rates, and insufflation temperature—to minimize the aerosolized dissemination of viable tumor cells (Peng et al., 2009).

Ultimately, rather than relying on uniform pedagogical methods, nursing managers must cultivate a highly supportive organizational climate that pairs targeted, approach-specific training with robust administrative oversight, thereby ensuring the seamless and high-fidelity translation of theoretical isolation protocols into everyday practice compliance.

## Limitations and Future Directions

This study has several limitations. First, due to time and budget constraints, it was conducted at a single hospital in western China, which limits the generalizability of the findings to other regions or healthcare settings. Second, the cross-sectional design employed convenience sampling, which may have resulted in a non-representative sample, reducing the external validity of the results, and preventing causal inferences.

Third, the data were collected using self-reported questionnaires, which may introduce social desirability bias, as participants might overestimate their positive attitudes or adherence to practices. To mitigate this inherent bias in future research, several methodological improvements should be implemented, such as adopting strictly anonymous data collection protocols, utilizing indirect questioning techniques, and combining self-reports with objective clinical audits or direct behavioral observations (triangulation approach).

Fourth, the statistical analysis was restricted to univariate tests without controlling for potential confounding variables. This methodological limitation may compromise the overall robustness and depth of our conclusions.

Therefore, future multi-center investigations utilizing randomized sampling and advanced multivariate analysis are strongly warranted to validate and extend our findings.

## Conclusions

In conclusion, this study elucidates the current baseline of operating room nurses’ regarding isolation techniques in oncological surgeries, successfully identifying specific vulnerabilities within each dimension. These findings offer valuable insights and clear guidance for developing future targeted intervention programs aimed at enhancing clinical competence in surgical tumor-free protection.

##  Supplemental Information

10.7717/peerj.21818/supp-1 Supplemental Information 1 English version blank questionnaire

10.7717/peerj.21818/supp-2 Supplemental Information 2 Data with numerical descriptions Containing the implications of numerical values

## Additional Information and Declarations

### Data Availability

The following information was supplied regarding data availability: The data is available in the Supplemental Files.
