# Supplementary material for: Knowledge, attitudes, and practices of Chinese operating room nurses toward isolation in oncologic surgery: a cross-sectional survey
Source: PeerJ. 2026 Nov 9;14:e21818. doi: 10.7717/peerj.21818 (PMC13757734; doi:10.7717/peerj.21818)
Supplement: Supplementary file 1 [file peerj-14-21818-s001.docx]

Supplementary Material

S1. Isolation Technique Knowledge–Attitude–Practice Questionnaire (Chinese–English Version)

Section 1. Demographic Characteristics

CN: 姓名

EN: Name

CN: 1. 性别（男/女）

EN: 1. Gender (Male/Female)

CN: 2. 年龄

EN: 2. Age

CN: 3. 您目前的层级（CN0–CN4）

EN: 3. Current Clinical Level (CN0–CN4)

CN: 4. 学历（专科/本科/硕士及以上）

EN: 4. Educational Level (Diploma/Bachelor/Master or above)

CN: 5. 职称（护士/护师/主管护师/副主任护师及以上）

EN: 5. Professional Title

CN: 6. 工作年限（<3年/3–5年/6–10年/≥11年）

EN: 6. Years of Nursing Experience

CN: 7. 手术室工作时间（<3年/3–5年/6–10年/≥11年）

EN: 7. Years of Operating Room Experience

Section 2. Knowledge (1 point for each correct answer)

Each item has one correct answer unless otherwise specified.

1. CN: 手术隔离技术是在外科手术中以防止或减少肿瘤细胞的脱落、种植和播散的技术。

EN: Surgical isolation techniques refer to procedures used during surgery to prevent or reduce tumor cell shedding, implantation, and dissemination.

2. CN: 下列术中切除肿瘤做法不正确的是。

EN: Which of the following intraoperative tumor resection practices is incorrect?

3. CN: 恶性肿瘤手术隔离手术的范围包括。

EN: The scope of isolation in malignant tumor surgery includes which of the following?

4. CN: 在手术中执行隔离技术后的操作包括。

EN: What procedures are included after implementing isolation techniques during surgery?

5. CN: 恶性肿瘤手术关于手术切口的保护下列哪项是错误的。

EN: Which of the following statements regarding incision protection in malignant tumor surgery is incorrect?

6. CN: 关于恶性肿瘤手术中隔离技术执行错误的是。

EN: Which of the following reflects incorrect implementation of isolation techniques during malignant tumor surgery?

7. CN: 卵巢癌术中瘤体切除后，手术隔离技术操作错误的是。

EN: After ovarian tumor resection, which isolation practice is incorrect?

8. CN: 在外科手术中，器械护士以下行为错误的。

EN: Which of the following behaviors of the scrub nurse is incorrect during surgery?

9. CN: 腹腔镜手术中，减少肿瘤细胞种植的气腹管理方式正确的是。

EN: Which pneumoperitoneum management approach is correct to reduce tumor implantation during laparoscopic surgery?

10. CN: 手术进行过程中再次使用手术刀时，洗手护士正确做法是。

EN: When reusing a scalpel intraoperatively, what is the correct action of the scrub nurse?

11. CN: 妇科手术术中严格按照无菌隔离技术进行,防止蜕膜组织和子宫内膜间质成分散落在手术区域。

EN: In gynecological surgery, strict sterile isolation prevents decidual and endometrial stromal tissue dissemination.

12. CN: 妇科手术中器械管理符合隔离技术的是。

EN: Which instrument management practice complies with isolation techniques in gynecological surgery?

13. CN: 以下哪项关于腹腔镜下宫颈癌无瘤防御手术操作描述正确的是。

EN: Which of the following describes correct tumor-free principles during laparoscopic cervical cancer surgery?

14. CN: 腔镜下内膜癌手术中无瘤防御操作描述正确的是（多选）。

EN: Which tumor-free practices are correct during laparoscopic endometrial cancer surgery? (Multiple choice)

15. CN: 妇科手术执行隔离技术的目的（多选）。

EN: What are the purposes of implementing isolation techniques in gynecological surgery? (Multiple choice)

16. CN: 外科手术时，接触哪些组织的器械、敷料均视为污染（多选）。

EN: During surgery, instruments and dressings contacting which tissues are considered contaminated? (Multiple choice)

Section 3. Attitudes (5-point Likert scale)

Response options: 1 = Strongly disagree; 2 = Disagree; 3 = Neutral; 4 = Agree; 5 = Strongly agree.

1. CN: 手术室护士应加强手术隔离技术知识的学习和培训。

EN: Operating room nurses should strengthen learning and training in surgical isolation techniques.

2. CN: 手术器械和敷料会携带肿瘤细胞或其他可种植细胞。

EN: Surgical instruments and dressings may carry tumor or other implantable cells.

3. CN: 护士的手术隔离技术配合对肿瘤手术患者的预后很重要。

EN: Nurses’ cooperation in isolation techniques is important for patient prognosis.

4. CN: 护士应该主动配合及提醒医生的隔离操作。

EN: Nurses should actively cooperate with and remind surgeons about isolation practices.

5. CN: 在术中按照手术隔离技术要求处置手术用物会影响手术的正常进程。

EN: Implementing isolation techniques may affect surgical workflow.

6. CN: 在术中对手术用物做隔离处理会增加护士的工作量。

EN: Isolation procedures increase nurses’ workload.

7. CN: 手术室护士都能很好地执行手术隔离技术。

EN: Operating room nurses can effectively implement isolation techniques.

8. CN: 对自己手术隔离技术知识掌握情况满意。

EN: I am satisfied with my knowledge of surgical isolation techniques.

9. CN: 执行手术隔离技术是护士的责任。

EN: Implementing isolation techniques is the responsibility of nurses.

10. CN: 执行手术隔离技术需要医生和护士的共同参与。

EN: Isolation techniques require collaboration between surgeons and nurses.

Section 4. Practice (5-point frequency scale)

Response options: 1 = Never; 2 = Rarely; 3 = Sometimes; 4 = Often; 5 = Always.

1. CN: 在开腹手术中使用标本袋或弯盘等隔离容器接取标本。

EN: Use specimen bags or curved trays as isolation containers during open surgery.

2. CN: 在腔镜手术中使用标本袋或弯盘等隔离容器接取标本。

EN: Use specimen bags or curved trays as isolation containers during laparoscopic surgery.

3. CN: 体腔内浸湿的纱布垫拧干之后再次使用（反向）。

EN: Reuse wrung-out gauze soaked inside the body cavity (reverse scored).

4. CN: 手套接触肿瘤或被肿瘤细胞污染的物品后未更换。

EN: Fail to change gloves after contact with tumor tissue.

5. CN: 接触过瘤体的器械未经消毒灭菌处理继续使用（反向）。

EN: Continue using instruments contacting tumor tissue without sterilization (reverse scored).

6. CN: 腹腔探查后提醒医生更换手套。

EN: Remind the surgeon to change gloves after abdominal exploration.

7. CN: 使用生理盐水、碘伏、蒸馏水处理接触过瘤体器械。

EN: Use saline, povidone-iodine or distilled water to manage tumor-contacted instruments.

8. CN: 恶性肿瘤手术中，关腹前更换手套并提醒医生更换手套。

EN: Change gloves and remind the surgeon before abdominal closure in malignant tumor surgery.

9. CN: 腔镜手术结束后规范气腹排气后再拔除套管针。

EN: Release pneumoperitoneum completely before trocar removal.

10. CN: 开腹手术中关闭腹腔前冲洗并加铺无菌巾。

EN: Irrigate and apply sterile drapes before abdominal closure.

11. CN: 肿瘤手术中，在器械台上划分隔离区域。

EN: Designate isolation zones on the instrument table.

Scoring Explanation

Knowledge items are scored as 1 for correct answers and 0 for incorrect answers. Multiple-choice items require all correct options to receive 1 point. Attitude and practice items are rated on 5-point Likert scales. Reverse-scored items are recoded prior to analysis. Higher total scores indicate better knowledge, more positive attitudes, and stronger adherence to isolation practices.
